# Development and validation of a nomogram for stratifying severe *Mycoplasma pneumoniae* pneumonia risk in children early

**DOI:** 10.3389/fped.2026.1927137

**Published:** 2026-10-02

**Authors:** Zhongying Chen, Wugao Ding, Zhengyan Gao

**Affiliations:** Department of Pediatrics, Second People's Hospital of Futian District, Shenzhen, China

**Keywords:** children, IL-6, nomogram, predictive model, severe *Mycoplasma pneumoniae* pneumonia

## Abstract

**Objective:**

This study aimed to develop and validate a clinically applicable diagnostic predictive model to facilitate early identification of severe *Mycoplasma pneumoniae* pneumonia (SMPP) within 24 h of admission.

**Methods:**

Clinical data from children with MPP hospitalized from January 2023 to December 2023, were retrospectively collected as the training set. Patients were classified into general MPP (GMPP) or SMPP groups based on whether SMPP occurred within 24 h of admission. After comparing the differences in clinical data between the two groups at admission, multivariate logistic regression was used to identify risk indicators for SMPP and construct a diagnostic predictive model. A nomogram was developed to visualize the model. Subsequently, data from children with MPP hospitalized between January 2024 and December 2024 were collected to conduct temporal external validation of the model. The predictive performance of the model was evaluated using receiver operating characteristic (ROC) curves, Hosmer-Lemeshow goodness-of-fit test, calibration curves, decision curve analysis (DCA), and clinical impact curves (CIC).

**Results:**

In total, 412 children with MPP were included, with 222 cases within the training set (119 SMPP, 53.60%) and 190 within the temporal external validation set (129 SMPP, 67.89%). Independent risk indicators for the occurrence of SMPP included the duration of fever before admission (OR = 2.019, 95% CI: 1.387–2.938), shortness of breath (OR = 111.599, 95% CI: 11.512–1,081.834), unresponsive to macrolide before admission (OR = 3.687, 95% CI: 1.529–8.892), interleukin-6 (IL-6) (OR = 1.052, 95% CI: 1.010–1.095), and pulmonary consolidation (OR = 31.634, 95% CI: 11.643–85.948) (all *P* < 0.05). The nomogram based on the five aforementioned variables demonstrated favorable discriminative performance, with areas under the ROC curve (AUC) of 0.916 (95% CI: 0.880–0.951) within the training set, 0.907 (95% CI: 0.873–0.946) within the internal validation, and 0.817 (95% CI: 0.758–0.876) within the temporal external validation set. The calibration curve and Hosmer-Lemeshow goodness-of-fit test showed that the model had good calibration. Both the DCA and CIC demonstrated favorable clinical utility of the model.

**Conclusion:**

The model developed in this study provides valuable reference for early risk stratification of pediatric SMPP and timely, individualized clinical decision-making in children with MPP. However, extensive external validation is required.

## 1 Introduction

*Mycoplasma pneumoniae* (MP) is a major pathogen causing community-acquired pneumonia (CAP) among children. Globally, epidemics of *Mycoplasma pneumoniae* pneumonia (MPP) occur every 3–7 years (1–4), accounting for >50% of pediatric hospitalizations for CAP during epidemic seasons. Following the COVID-19 pandemic, a global resurgence of MP infections emerged in 2023–2024. Reports from multiple countries [e.g., Germany (1), China (2, 3), Denmark (4), Switzerland (5), the UK (6), Spain (7), the USA (8), and other countries] indicate a significant rise in the incidence of pediatric MPP. Although MPP is typically mild and most children respond well to macrolide therapy, the incidence of severe MPP (SMPP) has risen recently (6–8), particularly in China (9–14). This trend may be attributable to rising prevalence of macrolide-resistant *Mycoplasma pneumoniae* (MRMP), heightened host immune-inflammatory responses, and increased co-infections. Various pulmonary and extrapulmonary complications caused by SMPP, such as plastic bronchitis, necrotizing pneumonia, and involvement of the neurological, circulatory, digestive, hematological, and mucocutaneous systems, might lead to life-threatening conditions and sequelae (15). These outcomes pose significant threats to health of children and increase healthcare burdens. Therefore, it is necessary to identify SMPP early and initiate effective treatment promptly. This might help children avoid or mitigate complications and sequelae, which is vital for improving prognosis.

Because epidemiological characteristics, clinical practice needs, and healthcare resource allocation vary across countries and regions, no unified diagnostic criteria for SMPP in children have been established worldwide. The *Chinese Consensus Guideline on Diagnosis and Treatment of MPP in Children* (15) proposes several diagnostic criteria for SMPP, including clinical manifestations (e.g., tachypnea, hypoxemia), abnormal chest imaging findings (e.g., x-ray or CT showing ≥2/3 consolidation of one lung or multi-lobar consolidation), and significant increases in inflammatory biomarkers [e.g., lactate dehydrogenase (LDH), D-dimer (D-D), C-reactive protein (CRP)]. The guideline states that cases are categorized as SMPP if patients meet any of the above criteria. However, these severity assessment criteria have not been systematically derived or clinically validated in the pediatric population. Previous studies suggest that the diagnostic performance of these individual markers is suboptimal (16, 17), and the optimal predictive thresholds for serological markers remain unclear. Furthermore, the clinical manifestations of SMPP in children are highly heterogeneous, and MPP can progress rapidly in children. Consequently, early identification of SMPP remains challenging. Owing to the absence of quantitative scoring for these risk indicators, it has been impossible to conduct individualized, precise quantification of the risk of SMPP in each child with MPP. Therefore, a quantitative scoring tool that can accurately and individually predict the risk of SMPP at an early stage needs to be developed. Recently, nomogram-based prediction models have been widely applied to assess the risk of pediatric diseases owing to their favorable predictive accuracy and simple, intuitive visualization (11–14). Nevertheless, nomograms specifically designed for the early diagnostic prediction of SMPP among children remain scarce. Moreover, the clinical utility and predictive performance of existing models have not been externally validated (11–13). Their generalizability is uncertain, and they are currently not suitable for dissemination as standardized assessment tools. This study aimed to investigate the clinical manifestations, laboratory indicators, and imaging characteristics of children with MPP in the early stages of the disease (within 1–5 days of fever), identify the independent risk indicators for the occurrence of SMPP, and integrate these multidimensional variables to construct a nomogram-based comprehensive model for early SMPP diagnosis within 24 h of admission. Moreover, the stability and generalizability of this model were assessed using internal validation and temporal external validation. These findings might provide a simple, rapid, and scientifically validated quantitative scoring tool for the early identification of SMPP among children and assist clinicians in detecting and treating SMPP during the critical early febrile phase (days 1–5), thereby reducing the occurrence of complications and sequelae.

## 2 Methods

### 2.1 Study subjects

Clinical data from children with MPP who were hospitalized in the Department of Pediatrics of the Second People's Hospital of Futian District, Shenzhen, between January and December 2023 were retrospectively collected to form the training cohort. Patients were classified into GMPP and SMPP based on whether SMPP occurred within 24 h of admission. Data from patients with GMPP and SMPP admitted to the same hospital between January and December 2024 were then gathered to create a temporal external-validation set. The inclusion and exclusion criteria for both the training set and temporal external validation set were the same. Inclusion criteria were as follows: (i) Age was 1–14 years; (ii) Diagnosis of MPP was based on the *Chinese Consensus Guideline on Diagnosis and Treatment of MPP in Children (2023)* (15). Children were diagnosed with MPP if they presented with: (a) symptoms encompassing fever, cough, wheezing, and shortness of breath; (b) abnormal findings on lung auscultation and/or chest imaging indicative of pneumonia; and (c) at least one of the following laboratory criteria: (d) Positive MP-DNA; (e) A single serum MP antibody titer ≥1:160 [particle agglutination (PA) method]; or (f) An increase of fourfold or more in the MP antibody titer in paired serum samples during the course of the disease; (iii) Duration of fever at admission was ≥24 and ≤120 h; (iv) Complete data on cases. Exclusion criteria were as follows: (i) Severe respiratory or circulatory system diseases, kidney or liver diseases, connective tissue diseases, and other underlying diseases; (ii) Long-term utilization of systemic corticosteroids or immunosuppressive drugs; (iii) Duration of fever was >120 h at admission or without a fever before admission; (iv) People who were allergic to azithromycin, erythromycin, and doxycycline; (v) Use of systemic steroids, human immunoglobulin, or other treatments before confirming progression to SMPP; (vi) Incomplete data on cases (people with >50% missing laboratory data or without x-ray or CT examination); (vii) Laboratory test or chest imaging examination >24 h of admission; (viii) Age was <1 year or >14 years. This study was approved by the Ethics Committee of the Second People's Hospital of Futian District, Shenzhen. As a retrospective study with anonymized data collection, informed consent was unnecessary. The number of patients initially assessed, the reasons for exclusion, and the final sample sizes for each set described above are visually presented in the flowchart (Figure 1).

**Figure 1:**
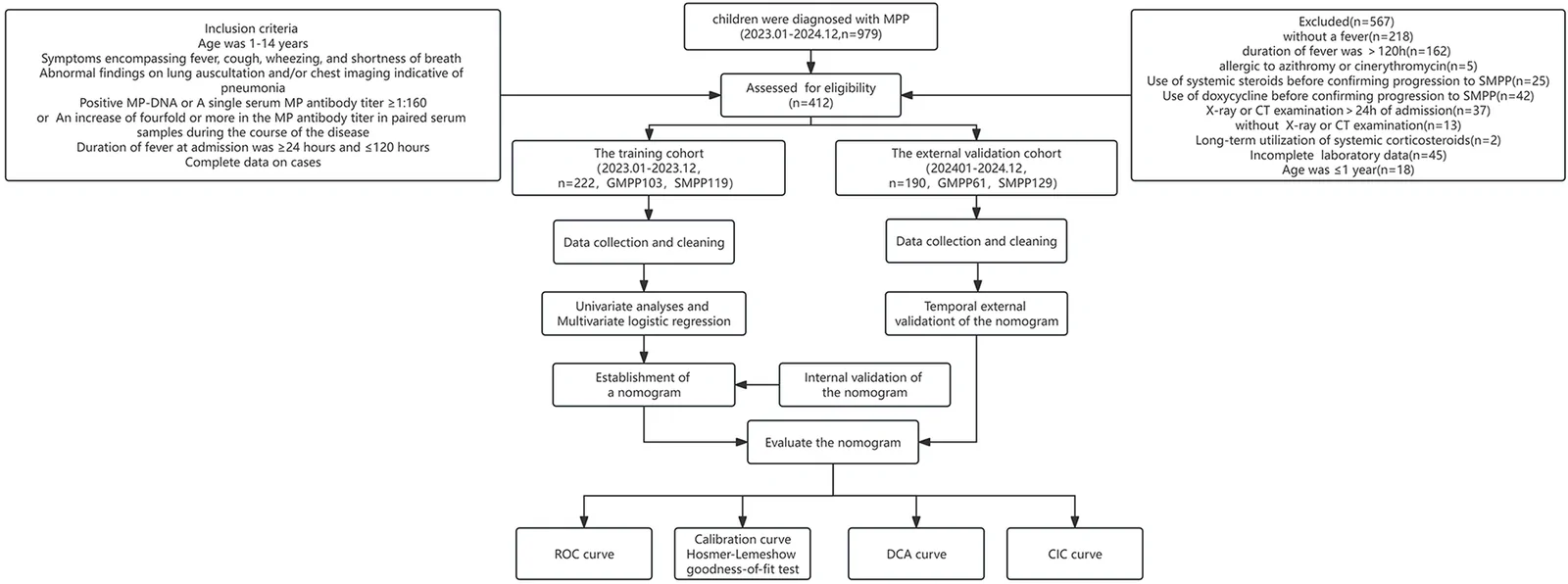
Flow chart of the study process.

### 2.2 Collection of clinical data

Clinical data of the children were collected through the electronic medical record system, encompassing demographic information, history of allergic diseases, clinical manifestations, laboratory tests [White blood cell count (WBC), neutrophil count (N), lymphocyte count (L), neutrophil percentage (N%), lymphocyte percentage (L%), neutrophil-to-lymphocyte ratio (NLR), hemoglobin (Hb), platelet count (PLT), platelet-to-lymphocyte ratio (PLR), CRP, procalcitonin (PCT), IL-6, lactate dehydrogenase (LDH), alanine aminotransferase (ALT), aspartate aminotransferase (AST), creatine kinase (CK), creatine kinase isoenzyme (CK-MB), uric acid (UA), albumin (ALB)], D-D, ferritin, respiratory pathogens (Influenza A Virus, Influenza B Virus, Parainfluenza Virus, Respiratory Syncytial Virus, Adenovirus, MP) identified by multiplex polymerase chain reaction (PCR), data derived from chest imaging, treatment before and after admission, and outcomes of patients. Clinical manifestations were derived from records during the initial course of the disease. Relevant laboratory and imaging indicators were obtained from tests conducted within 24 h of admission. If laboratory or imaging results were missing at admission, relevant test results obtained from outpatient examinations at the same hospital within 48 h prior to admission were used as substitutes.

### 2.3 Grouping scheme

Based on the *Chinese Consensus Guideline on Diagnosis and Treatment of MPP in Children (2023)* (15) and the Guidelines for the Diagnosis and Treatment of Community-Acquired Pneumonia in Children (2019 version) (18), children with MPP were classified into the SMPP group if they both met any of the following criteria within 24 h of admission and received additional corticosteroid therapy during hospitalization: (i) Persistent high fever (≥39 °C) for ≥5 days or ultra-hyperpyrexia; (ii) Shortness of breath and signs of increased work of breathing (moaning, nasal flaring, and suprasternal/intercostal/subcostal retractions); (iii) Presence of extrapulmonary complications; (iv) Oxygen saturation ≤93% without oxygen supplementation; (v) Chest imaging findings showing ≥2/3 consolidation of one lung or multi-lobar consolidation or the presence of pleural effusion; (vi) Progressive clinical symptoms with radiographic evidence of lesion progression, defined as a >50% increase in lesion size within 24–48 h. Patients who did not meet these criteria were included in the GMPP group.

### 2.4 Definition and assessment of variables

Shortness of breath was defined as a respiratory rate ≥40 breaths per minute in children aged 1–5 years and ≥30 breaths per minute in children aged >5 years (18). Unresponsive to macrolide was defined as no clinical or radiological improvement or further deterioration after 48–72 h of macrolide treatment in MPP cases (15, 19). Pulmonary consolidation was defined as homogeneous dense opacity in at least one lobe, with or without an air-bronchogram in the lesion (20).

### 2.5 Data preprocessing

First, patients were excluded if more than 50% of their laboratory test results were missing or if they lacked x-ray or CT examination results. Variables with >25% missing data were excluded, and multiple imputation was performed for all remaining covariates prior to statistical analysis to enhance methodological rigor and reduce potential selection bias. In the final dataset, we identified 11 variables from clinical manifestations, 9 variables from routine blood tests, 10 variables from biochemical detection data, 1 variable from respiratory pathogen tests, and 4 variables from imaging features.

### 2.6 Statistical analysis

All statistical analyses were conducted using R (version 4.4.3). A two-tailed *P* < 0.05 signified statistical significance. Categorical variables were described using percentages (%), and inter-group comparisons were conducted using the Chi-square test. Normally distributed continuous variables were presented as mean ± standard deviation, and inter-group comparisons were conducted using independent samples *t*-test. Non-normally distributed continuous variables were presented using the median and interquartile range (IQR), and inter-group comparisons were conducted using the Mann–Whitney *U* test. The variable differences were compared between the SMPP and GMPP groups in the training set. Variables with *P* < 0.1 were included in a multivariate logistic regression analysis to identify independent risk factors for SMPP. During this analysis, we used a probability threshold of 0.1 for exclusion and 0.05 for inclusion. Variance inflation factors (VIF) were used to analyze potential multicollinearity among the risk factors, and variables with a VIF >5 were excluded. A diagnostic predictive model that incorporated independent risk factors was constructed using logistic regression and then converted into a nomogram using the rms package. During the analysis, all continuous variables retained their original form and were not converted into categorical variables. Internal validation was performed using bootstrapping with 1,000 resamples. Clinical data from children with MPP admitted during a different time period from that of the training set were collected for temporal external validation of the model. The receiver operating characteristic (ROC) curve was plotted to estimate the discriminative capability of the model. The calibration curve and Hosmer-Lemeshow goodness-of-fit test were used to evaluate the prediction accuracy. Decision curve analysis (DCA) and clinical impact curve (CIC) were used to assess the clinical utility of the model (Figure 1).

## 3 Results

### 3.1 Basic characteristics of included cases

In total, 412 children were included. The median age was 6 years (IQR 4.6–8.0), and 224 (54%) were boys. The median hospital days were 6 (IQR: 5–7). Among them, the training set included 222 cases (103 within the GMPP group and 119 within the SMPP group) and the temporal external validation set included 190 cases (61 within the GMPP group and 129 within the SMPP group). Significant differences between the training set and temporal external validation set were observed regarding the duration from fever to the first receipt of macrolides, WBC, N, L, PLT, PCT, IL-6 and ALT (*P* < 0.05) (Table 1). The roles of the training and validation sets and the process of model development and validation are shown in the flowchart (Figure 1). During the data analysis, data on D-D and ferritin were excluded because these tests were not performed in more than 25% of the included children. Routine blood test data for 31 children (7.5% of the total cohort) and chest imaging data for 86 children (18.4% of the total cohort) were obtained from outpatient examinations conducted within 48 h before admission. All other data included in the analysis were obtained within 24 h of admission.

**Table 1:** The variables of the training and validation cohorts.

No	Variables	Training cohort (*n* = 222)	Validation cohort (*n* = 190)	All (*n* = 412)	*P*
1	Sex				0.451
	Female, *n* (%)	97 (44)	91 (48)	188 (46)	
	Male, *n* (%)	125 (56)	99 (52)	224 (54)	
2	Age [years, M (Q1, Q3)]	6 (4.58, 8)	6 (4.58, 8)	6 (4.58, 8)	0.581
3	Duration of fever before admission				0.088
	One day, *n* (%)	18 (8)	16 (8)	34 (8)	
	Two days, *n* (%)	22 (10)	13 (7)	35 (8)	
	Three days, *n* (%)	42 (19)	48 (25)	90 (22)	
	Four days, *n* (%)	79 (36)	48 (25)	127 (31)	
	Five days, *n* (%)	61 (27)	65 (34)	126 (31)	
4	Peak of fever [℃, M (Q1, Q3)]	39 (38.6, 39.5)	39 (38.6, 39.5)	39 (38.6, 39.5)	0.395
5	Duration of cough before admission [day, M (Q1, Q3)]	5 (4, 6)	5 (4, 6)	5 (4, 6)	0.520
6	Wheezing, *n* (%)				0.303
	No	183 (82)	148 (78)	331 (80)	
	Yes	39 (18)	42 (22)	81 (20)	
7	Shortness of breath, *n* (%)				0.561
	No	201 (91)	176 (93)	377 (92)	
	Yes	21 (9)	14 (7)	35 (8)	
8	Extrapulmonary complications, *n* (%)				1.000
	No	213 (96)	182 (96)	395 (96)	
	Yes	9 (4)	8 (4)	17 (4)	
9	Macrolide-unresponsive before admission, *n* (%)				0.084
	No	105 (47)	107 (56)	212 (51)	
	Yes	117 (53)	83 (44)	200 (49)	
10	Duration from fever to the first receipt with macrolides [day, M (Q1, Q3)]	3 (2, 4)	3 (2, 5)	3 (2, 4)	0.021
11	History of allergic disease, *n* (%)				0.364
	No	207 (93)	182 (96)	389 (94)	
	Yes	15 (7)	8 (4)	23 (6)	
12	WBC [×10^9^/L, M (Q1, Q3)]	6.75 (5.37, 8.2)	7.2 (6.02, 8.84)	6.9 (5.66, 8.45)	0.017
13	N [×10^9^/L, M (Q1, Q3)]	4.28 (3.02, 5.3)	4.42 (3.51, 5.41)	4.31 (3.24, 5.37)	0.066
14	L [×10^9^/L, M (Q1, Q3)]	1.76 (1.37, 2.39)	1.86 (1.49, 2.58)	1.81 (1.42, 2.48)	0.070
15	N% [%, M (Q1, Q3)]	62.1 (54.9, 69.68)	62.15 (53.82, 68.77)	62.1 (54.5, 69.43)	0.776
16	L% [%, M (Q1, Q3)]	27.2 (20.72, 34.88)	27.1 (21.83, 33.58)	27.1 (21.48, 34.5)	0.959
17	NLR [M (Q1, Q3)]	2.25 (1.53, 3.31)	2.27 (1.56, 2.98)	2.26 (1.55, 3.09)	0.842
18	Hb [g/L, (*x* ± *s*)]	123.89 ± 9.47	123.46 ± 9.21	123.69 ± 9.34	0.642
19	PLT [×10^9^/L, M (Q1, Q3)]	248.5 (205.25, 298.75)	258 (226, 324.75)	254 (213, 306.25)	0.034
20	PLR [M (Q1, Q3)]	141.73 (105.26, 184.07)	140.74 (106.14, 179.14)	141.33 (105.7, 180.16)	0.719
21	CRP [mg/L, M (Q1, Q3)]	12.09 (5.3, 24.48)	11.36 (4.92, 23.3)	11.57 (5.18, 24.26)	0.869
22	PCT [ng/mL, M (Q1, Q3)]	0.12 (0.09, 0.21)	0.11 (0.07, 0.17)	0.12 (0.08, 0.19)	0.006
23	IL-6 [ng/mL, M (Q1, Q3)]	12.03 (5.75, 18.68)	16.41 (9.61, 23.3)	14.65 (7.61, 20.63)	<0.001
24	LDH [U/L, M (Q1, Q3)]	267.02 (243.8, 301.71)	263.58 (236.51, 296.9)	266.08 (240.52, 298.04)	0.180
25	ALT [U/L, M (Q1, Q3)]	14 (12, 18)	12 (11, 16)	13 (11.75, 17)	<0.001
26	AST [U/L, M (Q1, Q3)]	29 (24, 34)	28.5 (24, 34)	29 (24, 34)	0.585
27	CK [U/L, M (Q1, Q3)]	100.5 (74, 138)	99 (78, 133)	99.5 (75, 136.25)	0.747
28	CK-MB [U/L, M (Q1, Q3)]	20 (16, 25)	18 (15, 21)	19 (16, 23)	<0.001
29	ALB [g/L, M (Q1, Q3)]	43.05 (41.73, 44.6)	43.1 (41.52, 44.85)	43.1 (41.6, 44.7)	0.856
30	Uric acid [μmol/L, M (Q1, Q3)]	262.82 (226.64, 305.7)	264 (221.38, 315.37)	263.93 (225.59, 307.35)	0.912
31	Co-viral infections, *n* (%)				0.918
	No	190 (86)	161 (85)	351 (85)	
	Yes	32 (14)	29 (15)	61 (15)	
32	Bronchopneumonia, *n* (%)				0.267
	No	19 (9)	10 (5)	29 (7)	
	Yes	203 (91)	180 (95)	383 (93)	
33	Pulmonary atelectasis, *n* (%)				0.286
	No	212 (95)	186 (98)	398 (97)	
	Yes	10 (5)	4 (2)	14 (3)	
34	Pulmonary consolidation, *n* (%)				0.267
	No	138 (62)	129 (68)	267 (65)	
	Yes	84 (38)	61 (32)	145 (35)	
35	Pleural effusion, *n* (%)				0.597
	No	221 (100)	188 (99)	409 (99)	
	Yes	1 (0)	2 (1)	3 (1)	
36	Total hospital days [M (Q1, Q3)]	6 (5, 8)	6 (5, 7)	6 (5, 7)	0.733
37	Category				0.004
	GMPP, *n* (%)	103 (46)	61 (32)	164 (40)	
	SMPP, *n* (%)	119 (54)	129 (68)	248 (60)	

### 3.2 Comparison of clinical, laboratory, and imaging indices between the SMPP and GMPP groups within the training set

Within the training set, the SMPP group had significantly higher duration of fever before admission (days), higher peak body temperature (°C), and elevated levels of CRP and IL-6 relative to the GMPP group. The SMPP group also demonstrated significantly higher frequencies of shortness of breath, extrapulmonary manifestations, unresponsive to macrolide, history of allergic disease, co-infection with other viruses, bronchopneumonia chest imaging, and pulmonary consolidation. Additionally, the levels of UA were decreased in the SMPP group compared to the GMPP group. All differences were statistically significant (*P* < 0.05) (Table 2).

**Table 2:** Univariate comparison of clinical, laboratory and radiological features between GMPP and SMPP group in the training cohort.

No	Variables	GMPP (*n* = 103)	SMPP (*n* = 119)	Training cohort (*n* = 222)	*P*
1	Sex				0.498
	Female, *n* (%)	48 (47)	49 (41)	97 (44)	
	Male, *n* (%)	55 (53)	70 (59)	125 (56)	
2	Age [years, M (Q1, Q3)]	6 (4.25, 8)	7 (5.33, 8.88)	6 (4.58, 8)	0.05
3	Duration of fever before admission[day, M(Q1,Q3)]	3.00 [2.00, 4.00]	4.00 [4.00, 5.00]	4.00 [3.00, 5.00]	<0.001
	One day, *n* (%)	16 (16)	2 (2)	18 (8)	
	Two days, *n* (%)	16 (16)	6 (5)	22 (10)	
	Three days, *n* (%)	26 (25)	16 (13)	42 (19)	
	Four days, *n* (%)	25 (24)	54 (45)	79 (36)	
	Five days, *n* (%)	20 (19)	41 (34)	61 (27)	
4	Peak of fever [℃, M (Q1, Q3)]	39 (38.5, 39.3)	39 (38.95, 39.5)	39 (38.6, 39.5)	0.008
5	Duration of cough before admission [day, M (Q1, Q3)]	5 (3, 7)	4 (4, 5)	5 (4, 6)	0.716
6	Wheezing, *n* (%)				0.204
	No	89 (86)	94 (79)	183 (82)	
	Yes	14 (14)	25 (21)	39 (18)	
7	Shortness of breath, *n* (%)				<0.001
	No	102 (99)	99 (83)	201 (91)	
	Yes	1 (1)	20 (17)	21 (9)	
8	Extrapulmonary complications, *n* (%)				0.004
	No	103 (100)	110 (92)	213 (96)	
	Yes	0 (0)	9 (8)	9 (4)	
9	Macrolide-unresponsive before admission, *n* (%)				0.018
	No	58 (56)	47 (39)	105 (47)	
	Yes	45 (44)	72 (61)	117 (53)	
10	Duration from fever to the first receipt with macrolides [day, M (Q1, Q3)]	2 (1, 4)	3 (2, 4)	3 (2, 4)	0.116
11	History of allergic disease, *n* (%)				0.015
	No	91 (88)	116 (97)	207 (93)	
	Yes	12 (12)	3 (3)	15 (7)	
12	WBC [×10^9^/L, M (Q1, Q3)]	6.73 (5.3, 8.98)	6.78 (5.4, 7.91)	6.75 (5.37, 8.2)	0.634
13	N [×10^9^/L, M (Q1, Q3)]	4.23 (2.84, 5.55)	4.28 (3.13, 5.1)	4.28 (3.02, 5.3)	0.859
14	L [×10^9^/L, M (Q1, Q3)]	1.77 (1.43, 2.55)	1.73 (1.33, 2.21)	1.76 (1.37, 2.39)	0.148
15	N% [%, M (Q1, Q3)]	61.4 (51.6, 68.6)	62.3 (56.05, 70.6)	62.1 (54.9, 69.68)	0.069
16	L% [%, M (Q1, Q3)]	27.3 (21.8, 36.75)	27 (19.7, 33.05)	27.2 (20.72, 34.88)	0.191
17	NLR [M (Q1, Q3)]	2.23 (1.47, 3.09)	2.31 (1.65, 3.54)	2.25 (1.53, 3.31)	0.265
18	Hb [g/L, M (Q1, Q3)]	123 (118, 131)	123 (117, 132)	123 (118, 131)	0.979
19	PLT [×10^9^/L, M (Q1, Q3)]	254 (208.5, 312)	247 (203.5, 289.5)	248.5 (205.25, 298.75)	0.129
20	PLR [M (Q1, Q3)]	135.17 (105.68, 178.19)	147.29 (104.67, 187.41)	141.73 (105.26, 184.07)	0.552
21	CRP [mg/L, M (Q1, Q3)]	8.42 (3.7, 17.75)	15.09 (6.92, 29.78)	12.09 (5.3, 24.48)	<0.001
22	PCT [ng/mL, M (Q1, Q3)]	0.12 (0.08, 0.19)	0.13 (0.09, 0.23)	0.12 (0.09, 0.21)	0.185
23	IL-6 [ng/mL, M (Q1, Q3)]	10.18 (4.02, 15.71)	13.81 (8.56, 21.88)	12.03 (5.75, 18.68)	0.001
24	LDH [U/L, M (Q1, Q3)]	258.55 (241.68, 298.15)	272 (247.88, 306.08)	267.02 (243.8, 301.71)	0.134
25	ALT [U/L, M (Q1, Q3)]	14 (12, 17)	14 (12, 18)	14 (12, 18)	0.564
26	AST [U/L, M (Q1, Q3)]	29 (24, 34)	28 (24, 34)	29 (24, 34)	0.657
27	CK [U/L, M (Q1, Q3)]	99 (74.5, 145.5)	101 (71.5, 134)	100.5 (74, 138)	0.975
28	CK-MB [U/L, M (Q1, Q3)]	21 (16.5, 25)	20 (16, 24)	20 (16, 25)	0.585
29	ALB [g/L, M (Q1, Q3)]	43 (41.8, 44.55)	43.1 (41.65, 44.7)	43.05 (41.73, 44.6)	0.854
30	Uric acid [μmol/L, M (Q1, Q3)]	269.81 (238.22, 322.97)	254.13 (215.56, 300)	262.82 (226.64, 305.7)	0.009
31	Co-viral infections, *n* (%)				0.03
	No	82 (80)	108 (91)	190 (86)	
	Yes	21 (20)	11 (9)	32 (14)	
32	Bronchopneumonia, *n* (%)				<0.001
	No	17 (17)	2 (2)	19 (9)	
	Yes	86 (83)	117 (98)	203 (91)	
33	Pulmonary atelectasis, *n* (%)				0.346
	No	100 (97)	112 (94)	212 (95)	
	Yes	3 (3)	7 (6)	10 (5)	
34	Pulmonary consolidation, *n* (%)				<0.001
	No	95 (92)	43 (36)	138 (62)	
	Yes	8 (8)	76 (64)	84 (38)	
35	Pleural effusion, *n* (%)				1
	No	103 (100)	118 (99)	221 (100)	
	Yes	0 (0)	1 (1)	1 (0)	
36	Total hospital days [M (Q1, Q3)]	6 (5, 7)	7 (6, 8)	6 (5, 8)	<0.001

### 3.3 Multivariate logistic regression analysis of independent risk indicators for SMPP

Though no significant differences were observed in age and N% between the two groups, the probability of significance was at the critical threshold (ranging from 0.05 to 0.1) (Table 2). Prior studies suggested that age and N% were predictive indicators for SMPP. Therefore, using the occurrence of SMPP as the dependent variable (coded as severe = 1, mild = 0), the indicators from the univariate analysis in Table 2 with *P* < 0.01 were included as independent variables in the multivariate logistic regression analysis. The results demonstrated that duration of fever before admission (days) (OR = 2.019, 95% CI: 1.387–2.938), shortness of breath (OR = 111.5999, 95% CI: 11.512–1,081.834), unresponsive to macrolide before admission (OR = 3.687, 95% CI: 1.529–8.892), IL-6 (OR = 1.052, 95% CI: 1.010–1.095), and pulmonary consolidation (OR = 31.634, 95% CI: 11.643–85.948) were independent risk indicators for SMPP (all *P* < 0.05, Table 3). The result of multicollinearity analysis showed that the VIF values for fever before admission (days), shortness of breath, unresponsive to macrolide before admission, IL-6, and pulmonary consolidation were 1.047, 1.102, 1.231, 1.086, and 1.169, respectively, indicating no obvious multicollinearity among these variables.

**Table 3:** Multivariate logistic regression for predicting SMPP in the training cohort.

Variables	*B*	Se	Wald	df	*P*	OR	OR (95% CI)
Duration of fever before admission(day)	0.703	0.191	13.462	1	0.000	2.019	1.387–2.938
Shortness of breath	4.715	1.159	16.551	1	0.000	111.599	11.512–1,081.834
Macrolide-unresponsive before admission (yes)	1.305	0.449	8.441	1	0.004	3.687	1.529–8.892
IL-6	0.051	0.021	6.005	1	0.014	1.052	1.010–1.095
Pulmonary consolidation (yes)	3.454	0.510	45.881	1	0.000	31.634	11.643–85.948
Constant	−5.312	0.938	32.039	1	0.000	0.005	

### 3.4 Construction of the nomogram

A nomogram for predicting SMPP was constructed based on duration of fever before admission (days), shortness of breath, unresponsive to macrolide before admission, IL-6, and pulmonary consolidation. In this model, for each additional day of pre-admission fever duration, the corresponding score increased by 14.28 points. The score for shortness of breath was 100 points. The score for unresponsive to macrolide was 31.2 points. For each 10-unit increase in IL-6, the score increased by 10.2 points. The score for pulmonary consolidation was 72.9 points (Figures 2, 3).

**Figure 2:**
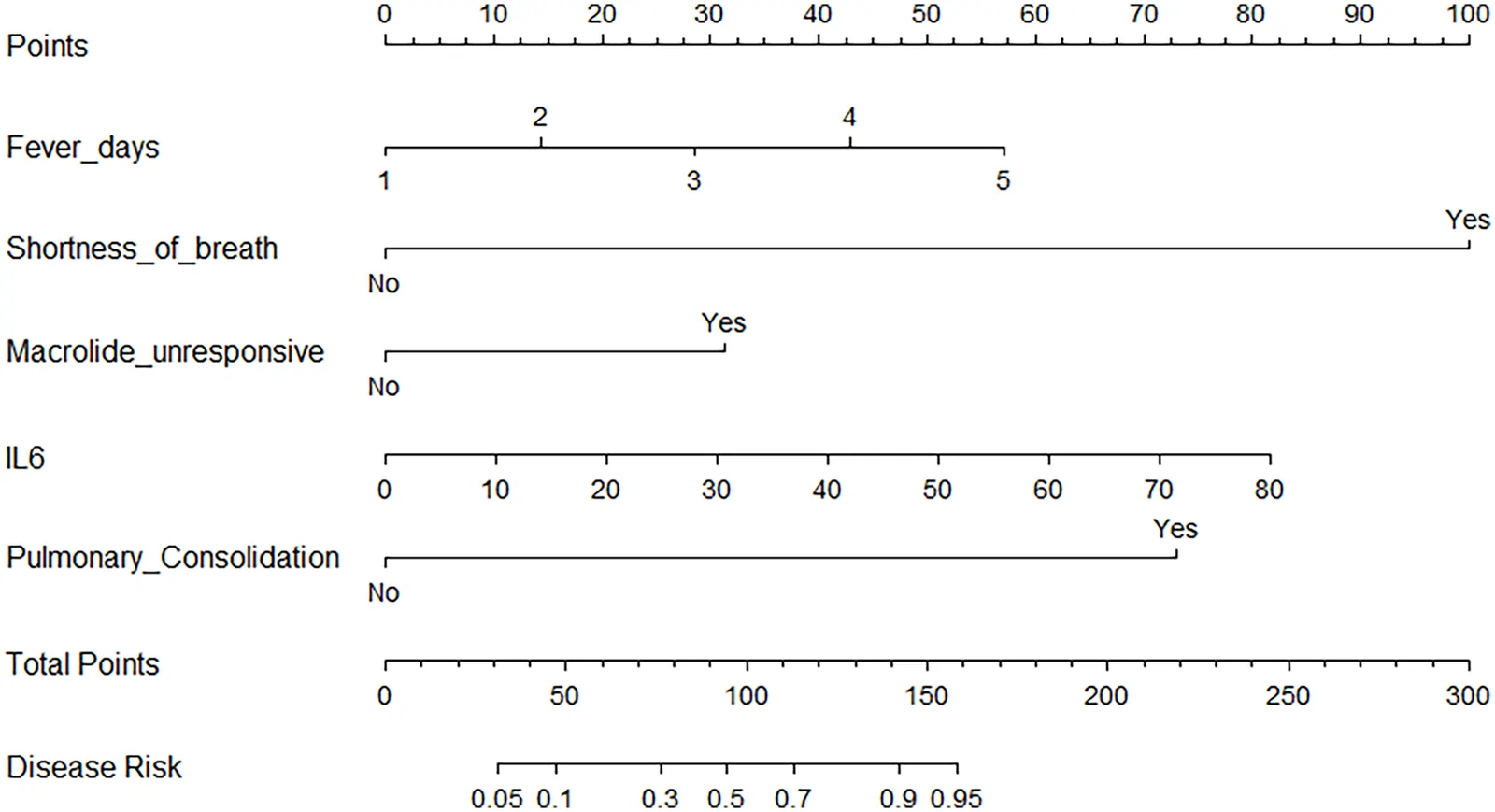
Nomogram prediction model for SMPP diagnosis based upon five independent predictors. The values of these included factors are marked on the corresponding axes. To acquire the corresponding scores for each variable, draw a vertical line upward to the “Points” axis. Sum the score for all predictors and locate the final value on the “Total Points” axis. Draw a line straight downward to the “Disease Risk” axis to determine the risk of SMPP.

**Figure 3:**
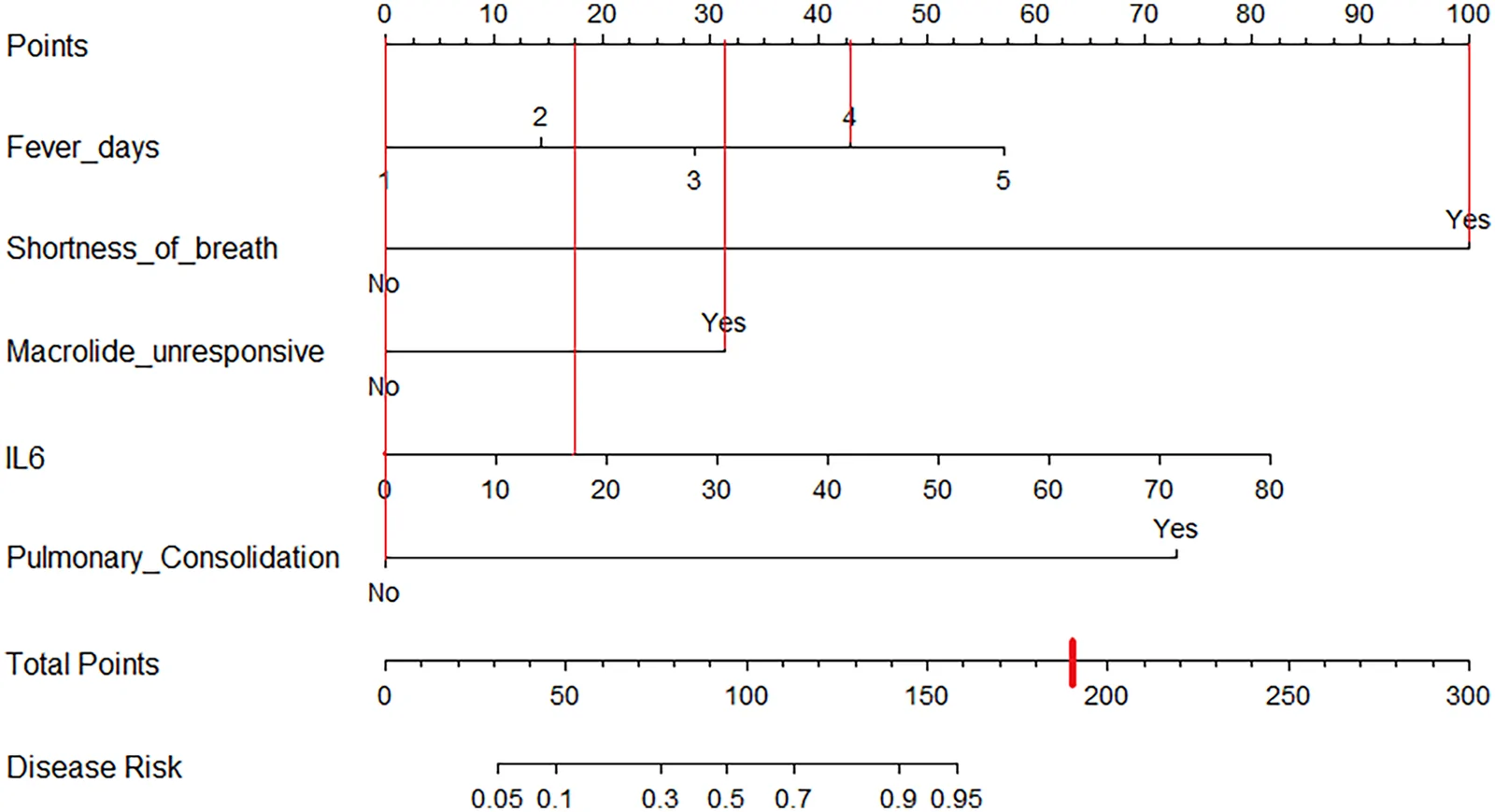
For example, a child with MPP presented with fever lasting 4 days prior to admission (42.9 points), accompanied by shortness of breath (100 points), showed macrolide-unresponsive before admission (31.2 points), had interleukin-6 level of 16.7 ng/mL at admission (17 points), and showed no pulmonary consolidation on chest imaging (0 points). The total points summed to 191.1. Based upon this nomogram, the predicted probability of developing to SMPP exceeds 95%.

### 3.5 Performance evaluation and validation of the model

The ROC curve demonstrated that the nomogram achieved an AUC of 0.916 (95% CI: 0.880–0.951) within the training set, 0.907 (95% CI: 0.873–0.946) within internal validation. This finding indicated good early discriminative capacity between GMPP and SMPP. The optimal cutoff probability was 0.553, with corresponding sensitivity and specificity of 0.782 and 0.903 (Figure 4). Within the temporal external validation set, the AUC of the constructed model was 0.817 (95% CI: 0.758–0.876). The optimal cutoff probability was 0.553, with corresponding sensitivity and specificity of 0.612 and 0.902 (Figure 4).

**Figure 4:**
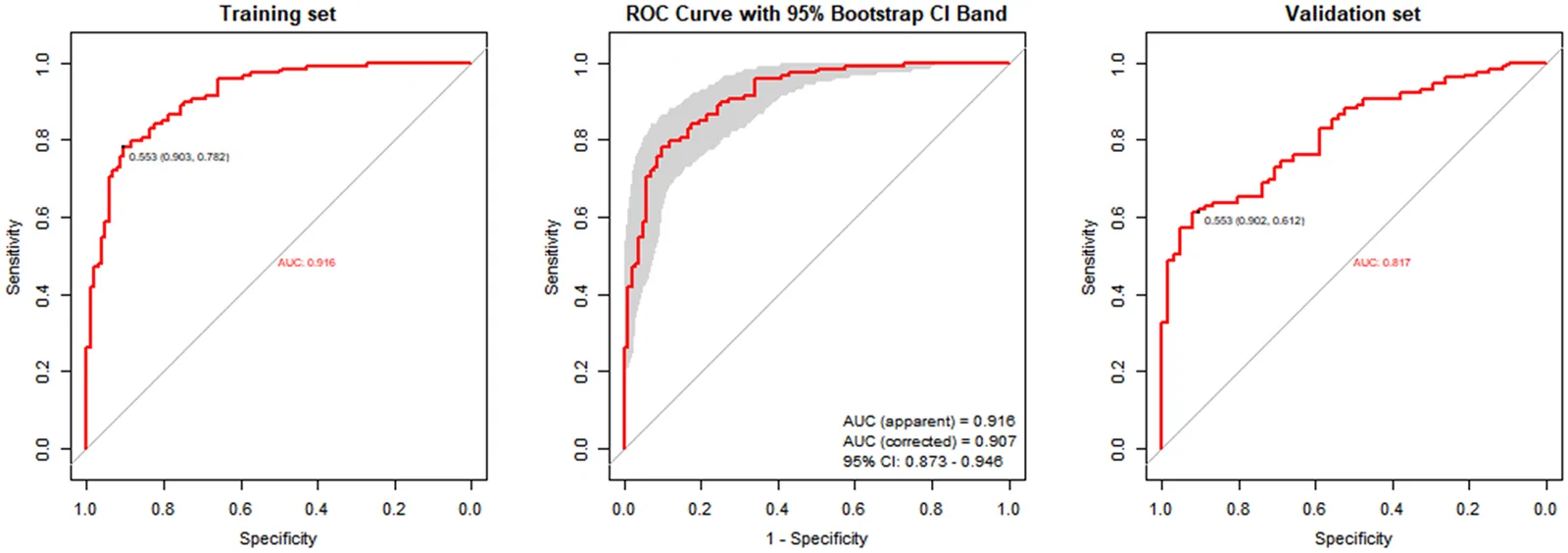
The ROC curves of the SMPP predictive nomogram in the training cohort and validation cohort. The AUC is 0.916 in the training cohort, and the AUC of the internal validation is 0.907 (95% CI: 0.873–0.946), indicating good discriminatory ability. The AUC is 0.817 in the validation cohort, suggesting a slight decrease in performance but still strong model accuracy.

The calibration curves of the model were plotted using bootstrapping with 1,000 resamples. In the training set, the calibration curve had an intercept of 0.005, a slope of 1.003, and a mean absolute error of 0.017. In the validation set, the corresponding values were 0.073, 0.836, and 0.037, respectively. Both calibration curves closely approximated the ideal line, indicating good agreement between predicted and observed probabilities (Figure 5). The Hosmer-Lemeshow goodness-of-fit test also indicated good calibration in the training set (*χ*^2^ = 5.082, *P* = 0.749) and the validation set (*χ*^2^ = 10.921, *P* = 0.206). These results demonstrated that the model had good calibration.

**Figure 5:**
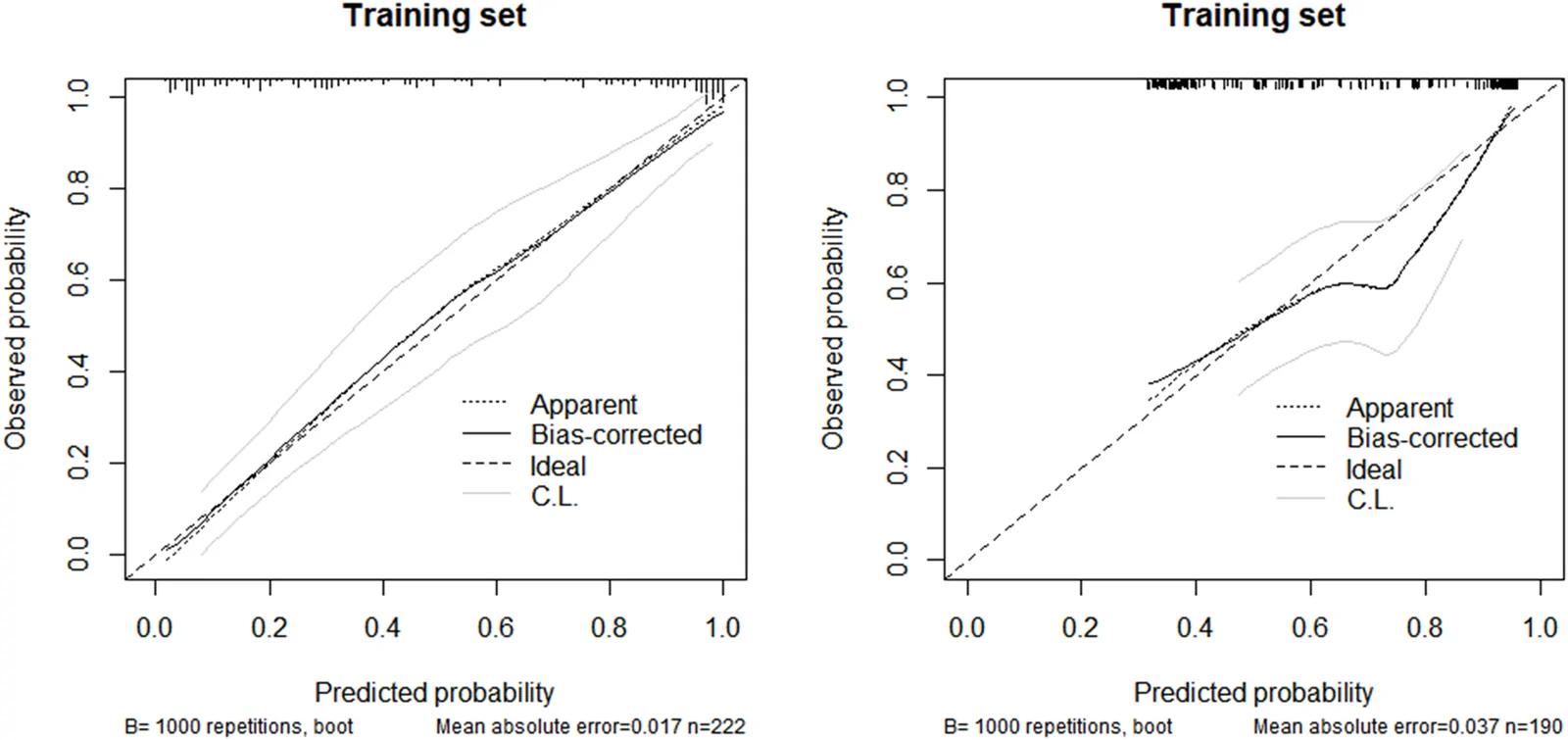
The calibration curves of the SMPP predictive nomogram in the training cohort and validation cohort.

The DCA curve showed that the nomogram provided substantial net benefit for guiding clinical intervention in children with SMPP across threshold probabilities of 10%–100% in the training set and 22%–100% in the temporal external validation set, indicating significant clinical utility in clinical decision-making (Figure 6). In particular, at the optimal cutoff probability of 0.553, the model showed a higher net benefit than at other cutoff values (Figure 6). The CIC revealed that the model could accurately identify patients who may benefit from intervention (Figure 7).

**Figure 6:**
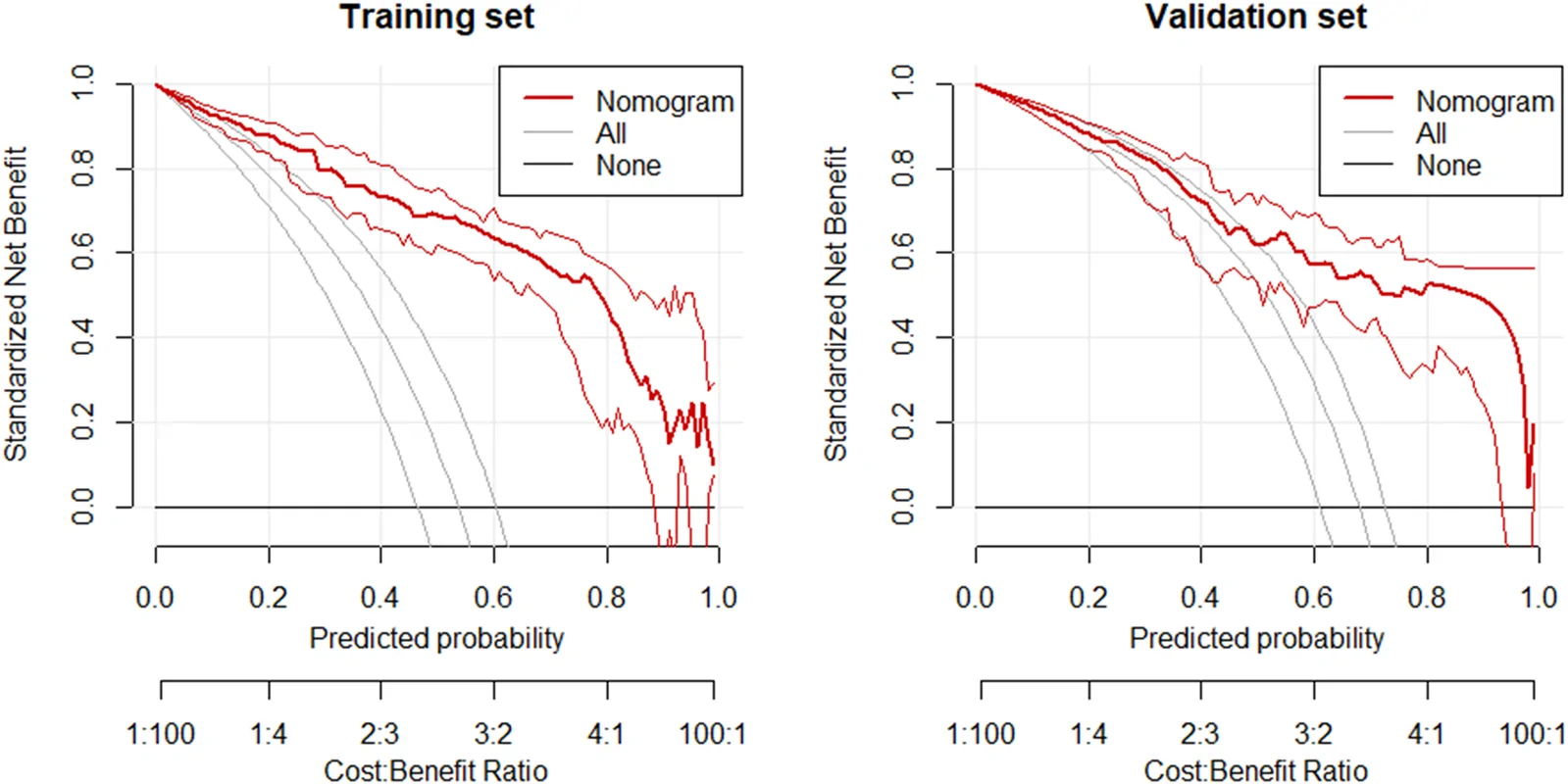
The DCA curves of the SMPP predictive nomogram in the training cohort and validation cohort. The *y*-axis represents the net benefit. The black solid line represents the assumption that all patients had GMPP. The gray solid line represents the assumption that all patients had SMPP. The red solid line represents the risk nomogram.

**Figure 7:**
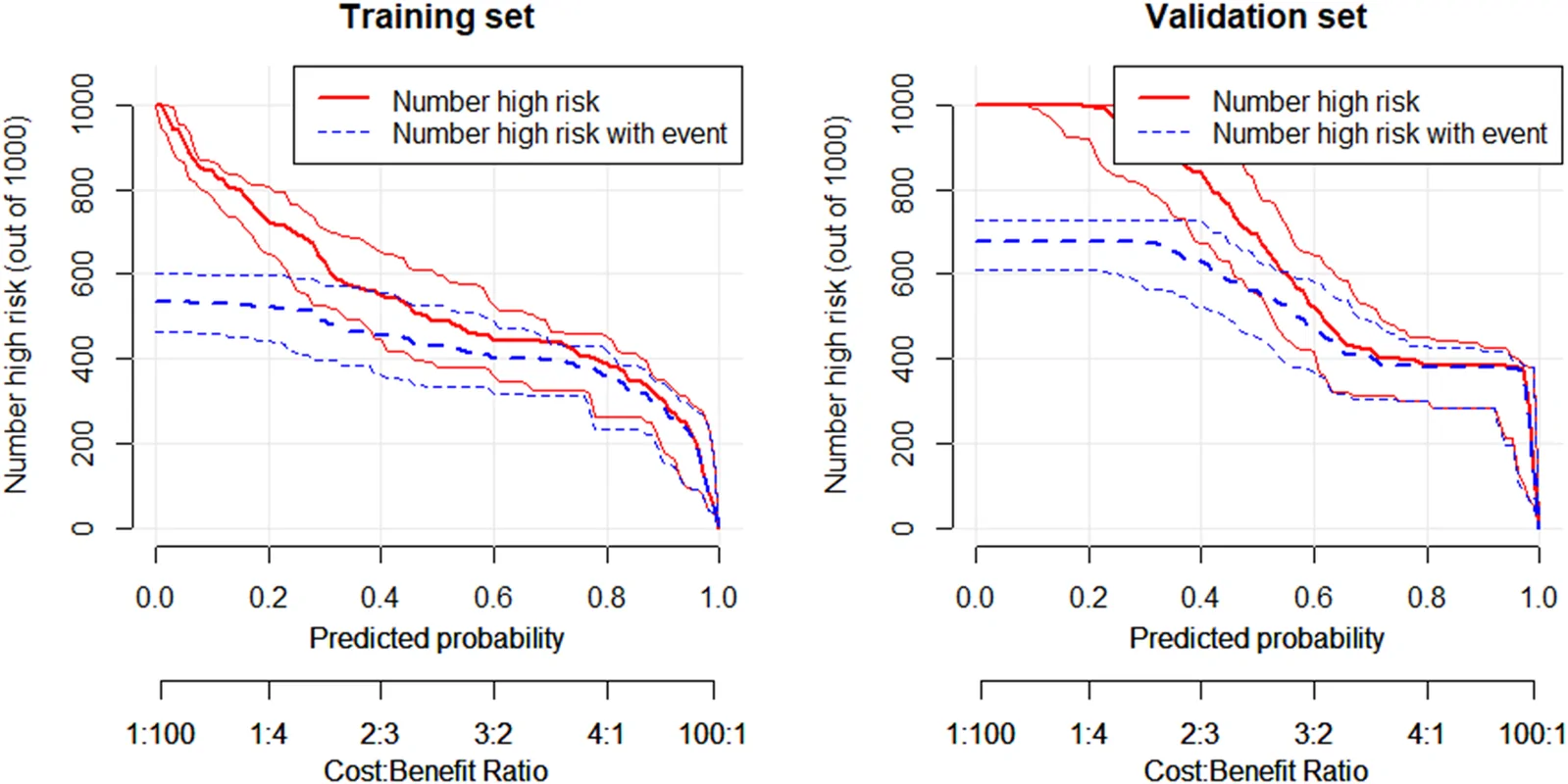
The CIC curves of the SMPP predictive nomogram within the training cohort and validation cohort. The red solid line represents the total number of individuals the model classified as “high-risk for SMPP” at different predictive probability thresholds; the blue dotted line represents the number of those classified as high-risk who actually suffered from SMPP. Applying the diagnostic model, the threshold probability for clinical decision-making is 0.553. Assuming that for every 1,000 cases of SMPP, at the threshold probability of 0.553, the number of individuals classified as high-risk for SMPP by the model within the training set is approximately 471, while the actual number of SMPP cases is 436; within the validation set, the number of individuals classified as high-risk for SMPP by the mode is approximately 608, while the actual number of SMPP cases is 532.

## 4 Discussion

MPP is typically mild and self-limiting. Nevertheless, some children might progress to SMPP, which generally occurs around 1 week after the onset of symptoms. SMPP typically arises owing to failed early identification or delayed treatment beyond the optimal post-fever window within 5–7 days. Early recognition is fundamental for initiating effective therapy. These findings further underscore the critical need for timely diagnosis. Following the relaxation of non-pharmaceutical interventions in the post-COVID-19 era, a high global incidence of MP infections has emerged. In several countries (particularly China), reports have observed a considerable elevation in the clinical severity of MPP in children, with a rising proportion of SMPP cases (6–14). A study from Zurich, Switzerland, reported that, compared with the pre-COVID-19 period, children with MPP hospitalized between April 2022 and May 2024 exhibited significantly higher rates of chest pain, pleural effusion, and obstructive lung disease (5). A multicenter retrospective study conducted in Spain analyzed the clinical data of 542 children with MPP hospitalized from January 2023 to March 2024. That study revealed that 62% of children with MPP developed to SMPP after admission and required supplemental oxygen, while 14% were admitted to the pediatric intensive-care unit (7). A retrospective study from Romania categorized hospitalized children with MPP into two cohorts (2018–2019 and 2023–2024) and found that, compared to the pre-COVID-19 period, children with MPP in 2023–2024 experienced more severe progression of disease, with higher rates of pulmonary consolidation, gastrointestinal complications, and dyspnoea (21). Our study reviewed the clinical records of 420 children hospitalized for MPP in Shenzhen, China, during the 2023–2024 and observed that the proportion of SMPP cases reached 60.2%. This finding supports the observed trend toward more severe illness in Chinese children with MP infections after 2023. This shift in the epidemiology and clinical presentation of pediatric MPP indicates that delays in the diagnosis and treatment of SMPP must be addressed promptly. Given the high incidence of SMPP, pediatricians need to adopt updated diagnostic and therapeutic approaches in clinical practice. Furthermore, it is essential to heighten early vigilance for SMPP in children at an early stage. Following comprehensive assessment with validated scoring tools, personalized and precise treatment should be implemented based on the severity of disease to reduce the complications and mortality of SMPP in children. However, to date, no validated and widely used comprehensive predictive model that integrates clinical manifestations, laboratory indicators, and imaging characteristics has been established for pediatric SMPP. In this study, we developed and preliminarily validated a nomogram model incorporating five easily accessible variables. This model provides clinicians with a simple, clinically applicable visual tool for early diagnosis of SMPP in children within 24 h of admission.

The early clinical symptoms of SMPP are non-specific and might progress rapidly. The *Chinese Consensus Guideline on Diagnosis and Treatment of MPP in Children (2023)* (15) indicates that children with MPP who exhibit shortness of breath within the first 5 days of illness or persistent high fever ≥5 days, or fail to respond to 72-hour macrolide therapy from onset, should be considered at risk for SMPP. The results of our study support this perspective. This study showed a significantly higher proportion of shortness of breath in SMPP. The odds ratio (OR) was 111.599 (95% CI: 11.512–1,081.834), and the relative risk was higher relative to other four predictive factors. This finding suggests that shortness of breath might be the most valuable early clinical predictor of SMPP. However, previous study have shown that, shortness of breath demonstrates only 47% sensitivity for diagnosing SMPP (16). Combined with the findings of this study, shortness of breath as a single diagnostic indicator has a low sensitivity and a high specificity for diagnosing SMPP in children. Fever is a common clinical symptom of MPP. Prolonged fever can induce systemic immune dysfunction, compromising the management of infection and inflammation, triggering an excessive immune-inflammatory response, and thereby promoting the occurrence and progression of SMPP (22). Prior studies have compared the duration of fever among children with GMPP and SMPP and found that prolonged fever is a risk indicator for SMPP in children (10–13, 22). This study also observed that children in the SMPP group demonstrated prolonged fever periods before admission relative to the GMPP group, with a higher proportion of patients experiencing fever for 5 days before admission. Multivariate logistic regression further confirmed that the duration of fever before admission is an independent predictive factor for SMPP (OR = 2.019, 95% CI: 1.387–2.938, *P* < 0.05).

Recently, emergence of macrolide-unresponsive MPP (MU-MPP) has complicated the management of MPP. MU-MPP is defined as MPP in which fever persists, and clinical signs and chest imaging show no improvement or further deterioration despite 48–72 h of macrolide therapy (15, 19). The primary cause of MU-MPP is the acquisition of resistance by MP through mutations in the 23S rRNA gene. Reduced susceptibility to macrolides leads to higher bacterial loads and a more intense immune-inflammatory response, resulting in more severe outcomes of diseases (10, 23). A study (23) in 2024 reported that, relative to children with macrolide-sensitive MPP, children with MU-MPP showed significantly prolonged duration of fever, a higher proportion of shortness of breath (23.61%) and hypoxemia (29.17%), and a higher proportion of progression to SMPP (52.78% vs. 34.83%; *P* < 0.05). This observation aligns with our findings. In our study, relative to the GMPP group, the SMPP group had an elevated proportion of unresponsive to macrolide before admission (61% vs. 44%, *P* < 0.05). The diagnosis of MU-MPP might serve as an early predictive indicator for SMPP (OR = 3.687, 95% CI: 1.529–8.892).

After entering the bloodstream, MP stimulates immune cells, airway epithelium, and pulmonary fibroblasts to produce IL-6. Elevated levels of serum IL-6 are detectable among children with MPP early in the infection and induce an excessive immune-inflammatory response in the body, continuously invading the lungs and respiratory tissues, inducing poor amelioration of symptoms, and thereby exacerbating the disease (22). Several studies have shown that IL-6 can be used to estimate the severity of disease among children with MPP and serves as an early predictive marker for SMPP (22, 24). However, the optimal cutoff value of IL-6 for predicting SMPP remains underexplored. The results of this study indicate that an IL-6 level >8.625 ng/mL measured within 1–5 days of fever can facilitate early identification of SMPP in children with MPP.

Chest imaging among children with MPP varies based on the severity of their condition. These manifestations might include bronchopneumonia, pulmonary consolidation, atelectasis, pleural effusion, and mosaic signs in the lungs. The severity of chest imaging findings is strongly linked to the clinical characteristics and outcomes of MPP. It might serve as an important indicator for prognosis (20, 25, 26). The study by Chu et al. (25) reported that low-dose chest CT has superior sensitivity and specificity relative to chest x-rays in diagnosing SMPP. Pulmonary consolidation is a stable and reliable CT feature, and quantitative analysis of lobar consolidation can comprehensively and accurately predict the progression of MPP in children. For suspected SMPP cases, low-dose chest CT may contribute to early identification. The study by Mu et al. (26) and Huang et al. showed that MPP children with pulmonary consolidation may develop complications such as plastic bronchitis, necrotizing pneumonia, and bronchiolitis obliterans, which increases the risk of progression to SMPP or refractory MPP (20, 25, 26). Several prior studies have established that pulmonary consolidation can serve as an early warning indicator for SMPP (10, 11, 20, 25, 26). This is possibly owing to an abnormal immune response induced by MP infection, which impairs airway immune function, causes ciliostasis, induces a marked diminishment in mucociliary clearance, induces the formation of mucus plug, atelectasis, and other changes, and severely hinders the absorption of pulmonary inflammation. In a study of 562 hospitalized children with MPP (11), pulmonary consolidation was observed on chest imaging in 69% of the SMPP group relative to 26% of the non-SMPP group (*P* < 0.01). LASSO regression analysis identified pulmonary consolidation as a significant factor in the progression of SMPP among children. This observation aligns with our findings. This study performed chest x-rays on all children and conducted low-dose chest CT scans on those with a high suspicion of SMPP at admission. The results showed that the incidence of pulmonary consolidation was significantly elevated in the SMPP group relative to the GMPP group (64% vs. 8%, *P* < 0.001), with an OR of 31.634 (95% CI: 11.643–85.948). The relative risk was solely second to shortness of breath, suggesting that pulmonary consolidation is an important predictive indicator for the occurrence of SMPP.

Currently, high-resolution chest CT (HRCT) remains the gold-standard imaging standard for diagnosing SMPP. However, radiation exposure limits its use in early diagnosis, longitudinal monitoring of treatment response, and long-term follow-up of pediatric lung disorders. Therefore, exploring non-invasive, radiation-free, convenient, and repeatable diagnostic and monitoring imaging tools is clinically important in pediatric patients. Lung ultrasound can assess the size of pulmonary consolidation and the presence of pleural effusion (27, 28). As a new, ionizing-radiation-free, safe, effective tool for imaging the lungs, it is increasingly used in clinical practice to evaluate the severity of MPP (27, 28). Compared to chest x-rays, chest CT, and lung ultrasound, lung magnetic resonance imaging (MRI) is free of ionizing radiation and provides multi-parameter imaging of the lung microstructure. It also allows for the visual and quantitative assessment of key lung functions, including ventilation, perfusion, and gas exchange, through the use of free-breathing proton and hyperpolarized gas MRI (29–31). In particular, hyperpolarized ^12^⁹Xe MRI is highly sensitive to regional lung functional abnormalities and is highly repeatable, making it well suited for precise assessment of lung disorders and for monitoring treatment efficacy in pediatric patients (32, 33). In recent years, with the rapid advancement of technologies such as hyperpolarized gas MRI, lung MRI has been successfully applied to diagnose and manage lung disorders in children and neonates, including bronchopulmonary dysplasia, cystic fibrosis, and asthma (34). This finding demonstrates that lung MRI is a potentially important tool for assessing and monitoring pediatric and neonatal lung disorders. However, the application of lung MRI in pediatric CAP in real-world clinical settings has not been reported. This may be due to the lengthy duration and high cost of MRI examinations, particularly the limited accessibility of hyperpolarized gas lung MRI. Because techniques such as ultra-short echo time (UTE) sequences and hyperpolarized gas contrast imaging are limited in our centre, this study could not use lung MRI to assess abnormal changes in pulmonary structure and function in children with MPP. However, recent translational advances in functional lung MRI have opened new avenues for non-invasive, radiation-free assessment of regional ventilation in children. Ariyasingha (35) demonstrated the feasibility of millimeter-scale ventilation MRI using parahydrogen-hyperpolarized propane-d6 gas, achieving a spatial resolution of 1 mm × 1 mm × 9 mm on a clinical 0.35T open MRI scanner. This approach utilizes proton-hyperpolarized gases that are inexpensive to produce and can be detected on virtually any clinical MRI scanner without hardware modification, potentially overcoming the cost and accessibility barriers of hyperpolarized ^12^⁹Xe. In a related study (36), the same group developed hyperpolarized butane gas with a nearly doubled hyperpolarized state lifetime (T1 ≈ 1.6 s). It demonstrated subsecond 2D ventilation imaging with 1.6 mm × 1.6 mm in-plane resolution in excised rabbit lungs. Although these techniques remain in the preclinical stage, they offer a promising path toward rapid, radiation-free, high-resolution functional lung imaging that could complement pulmonary physiological assessment in pediatric pneumonia. Future studies are needed to evaluate their clinical utility in early risk stratification of SMPP.

A nomogram is a visual predictive tool that integrates multiple predictors. Based on the nomogram, the corresponding score for each predictive factor is obtained. The total score is calculated by summing these individual scores. The predictive probability corresponding to the total score represents the likelihood of an individual experiencing the outcome event. This provides a specific score for the risk of disease occurrence in the individual and serves as a quantitative assessment tool for clinical evaluation. Prior studies have attempted to establish nomogram-based prediction models for predicting SMPP in children. Li et al. (12) developed a nomogram-based prediction model that includes eight independent risk indicators (age, decreased breath sounds, respiratory rate, duration of fever, hospital stay, co-infection with other pathogens, levels of ferritin, and levels of LDH) with an AUC of 0.862 on internal validation. Zhang et al. (13) constructed the first dynamic nomogram for predicting SMPP based on factors such as age, AGR, NLR, CRP, ESR, MPV, co-infection, pleural effusion, primary disease, fever duration ≥7 days, and wheezing. The AUC of that model was 0.867 within the training cohort and 0.840 within the validation cohort. Though the predictive performance of these models was acceptable in internal validation, none underwent external validation in an independent cohort. Consequently, their stability remains unevaluated, limiting generalizability and clinical applicability of the models. Furthermore, the model by Li et al. (12) includes the number of hospital days as a predictive factor, which is not suitable for early prediction of SMPP. In contrast, the model by Zhang et al. (13) is relatively complex, with 11 predictive factors. The factor of co-infection is difficult to rapidly and accurately obtain in general medical institutions, posing challenges for its practical clinical application. Our study has numerous strengths relative to prior studies. First, the study was rigorously conducted. After constructing the model, internal validation was performed using bootstrapping with 1,000 resamples, and temporal external validation was conducted using an independent cohort of 190 children with MPP. The effectiveness of the model was evaluated using multiple metrics, including AUC, calibration curves, Hosmer-Lemeshow goodness-of-fit, DCA curves, and CIC. The model also demonstrated favorable diagnostic predictive capability in the independent external dataset, preliminarily confirming its generalizability and higher reliability. In addition, the predictive factors incorporated in this model are simpler and more comprehensive, covering clinical manifestations, laboratory indicators, and imaging data. These factors are low-cost and easily accessible, enhancing the generalizability of the model. More importantly, previous SMPP prediction models were typically built on data collected throughout the entire disease course, resulting in delayed diagnosis. Nevertheless, the predictive factors in this model are based on early clinical data from the first 1–5 days of fever among children with MPP. This finding contributes to the accurate prediction of risks of SMPP within the first 1–5 days of fever, facilitating earlier detection of pediatric SMPP and enabling earlier appropriate treatment and intervention.

In this study, the nomogram showed lower discriminative ability in the validation set (AUC = 0.817; 95% CI: 0.758–0.876) than in the training set (AUC = 0.916; 95% CI: 0.880–0.951). This discrepancy is partly due to the limited sample size of the training set, which may have led to overfitting. Additionally, because the temporal external validation set was collected at a later time period than the training set, the epidemiological characteristics of MPP had changed. A study conducted in China showed that since September 2023, there had been a significant increase in pediatric MPP cases with severe clinical manifestations, which were predominantly caused by MRMP (37). During the validation of the model, it was found that the disease composition in the validation set (a significantly higher proportion of SMPP, 67.89% vs. 53.60%) and certain predictive factors (such as the duration from fever to the first receipt of macrolides, WBC, and PLT) differed from those in the training set. Although these differences reflect the heterogeneity of real-world clinical data, they may affect the generalization performance of the model. Nevertheless, the model developed in this study demonstrated good discriminative ability, good calibration, and favorable clinical utility in the validation set, indicating that the model had relatively good generalizability within our center.

However, this study also has several limitations. First, it is a single-center retrospective study with a relatively small sample size. Children with incomplete clinical data were excluded, which might induce selection bias. Second, despite the implementation of rigorous internal and temporal external validation, the relatively small sample size may lead to an overestimation of the performance of the model. Furthermore, since the sample data were drawn from a single medical center and extensive geographic external validation across different medical centers was lacking, the validity and generalizability of the model have not been fully validated. This limitation restricts the dissemination of the model. Future studies should involve multi-center collaborations to enhance the diversity of populations and prospectively collect large-scale clinical data. It is necessary to conduct more extensive external validation and further refine the prediction model to enhance its generalizability. Third, several potentially predictive variables (such as levels of D-D, ferritin and positive MP resistance gene tests) were not included because >25% of the results for these variables were unavailable, which might have affected the performance of the model. In the future, new diagnostic technologies and indicators should be considered, such as radiation-free lung ultrasound for assessing pulmonary consolidation and pleural effusion, hyperpolarized gas MRI for assessing pulmonary structural and functional abnormalities, and novel biomarkers including serum transforming growth factor-β1. Additionally, more predictors with potential value should be incorporated to enhance the diagnostic accuracy and sensitivity of the model. Fourth, reliance on static biomarkers measured within 24 h of admission fails to capture dynamic trends. Consequently, it is not feasible to reveal the dynamic changes in these biomarkers or their temporal association with disease progression. Future studies incorporating longitudinal biomarker monitoring would help enhance the clinical utility of this model.

## 5 Conclusion

In conclusion, this study constructs a simple and intuitive early assessment nomogram-based model for SMPP in children, based on duration of fever before admission (days), shortness of breath, unresponsive to macrolide before admission, levels of IL-6, and pulmonary consolidation. Multidimensional performance evaluation and preliminary validation support the strong discriminative capability, excellent calibration, robust clinical utility, relatively good generalizability of the model in our center. The model enables clinicians to quantitatively estimate the probability of SMPP in children during the critical early febrile phase (days 1–5). This capability is potentially useful for early risk stratification of pediatric SMPP and may help fulfill the clinical objective of early diagnosis of SMPP within 24 h of admission. Future prospective multicenter clinical trials are required to validate its practical utility in clinical decision-making across diverse populations and medical centers. Once thoroughly externally validated, this nomogram model might serve as an auxiliary tool for clinicians to identify SMPP at an early stage, thereby facilitating individualized risk assessment, rapid clinical stratification, and precise treatment within 24 h of admission for children with MPP.

## Data Availability

The original contributions presented in the study are included in the article/Supplementary Material, further inquiries can be directed to the corresponding author.
